# Sildenafila como Terapia Adequada de Transplante Cardíaco para Insuficiência Cardíaca Avançada Associada à Hipertensão Pulmonar Fixa

**DOI:** 10.36660/abc.20200631

**Published:** 2021-02-19

**Authors:** Pedro Schwartzmann

**Affiliations:** 1 Hospital Unimed Ribeirão PretoRibeirão PretoSPBrasilHospital Unimed Ribeirão Preto, Ribeirão Preto, SP - Brasil; 2 Centro de PesquisaCentro Médico RBSRibeirão PretoSPBrasilCentro de Pesquisa (CAPED) - Centro Médico RBS, Ribeirão Preto, SP - Brasil

**Keywords:** Insuficiência Cardíaca, Hipertensão Pulmonar, Sildenafila/uso terapêutico, Citrato de Sildenafila/uso terapêutico, Transplante Cardíaco

O transplante cardíaco (TC) tem sido associado a melhora significativa na sobrevida e na qualidade de vida em pacientes com insuficiência cardíaca (IC) avançada. Apesar de um aumento considerável na indicação de dispositivos de assistência ventricular esquerda, o TC ainda permanece a abordagem padrão-ouro neste contexto clínico.^1^ Embora seja considerado o tratamento padrão-para IC avançada, vários motivos têm prejudicado o uso disseminado do TC. A disponibilidade de doadores é limitada em comparação com o número crescente de receptores potenciais. Além disso, a maior expectativa de vida dos pacientes devido à melhor farmacoterapia da IC tem desafiado a contraindicação relacionada à idade para o procedimento, bem como a presença de mais comorbidades ligadas ao envelhecimento.^2^

Outro ponto a ser observado relacionado à candidatura ao TC é a hipertensão pulmonar (HP), que está presente em mais de 60% dos pacientes com IC com fração de ejeção reduzida (ICFEr) e em mais de 54% dos pacientes com IC com fração de ejeção preservada (ICFEp) e também pode ser responsável por até 50% das complicações pós-TC.^1^ Uma resistência vascular pulmonar elevada (RVP)> 2,5 unidades Wood está associada a um aumento de quase 30% na mortalidade no primeiro mês após o transplante.^2^ Essa HP associada à IC é considerada o resultado do efeito passivo do aumento da pressão diastólica final do ventrículo esquerdo, juntamente com a vasorreatividade secundária à vasoconstrição e remodelamento pulmonar arterial.^3,4^ Quando não é reversível com o teste de reatividade (*challenge test*) ao vasodilatador, a HP tem sido fortemente associada à disfunção ventricular direita, hospitalizações por IC, piora na qualidade de vida e redução da sobrevida.^5^ As diretrizes da *International Society for Heart and Lung Transplantation* consideram a presença de HP grave pré-transplante como uma contraindicação relativa ao transplante cardíaco.^6^ Da mesma forma, a desproporcionalidade entre a necessidade de transplante cardíaco e a reduzida disponibilidade de doadores deve levar a uma seleção ainda mais criteriosa dos potenciais candidatos ao TC e novas tentativas de redução da hipertensão pulmonar pré-TC são necessárias.

Uma das abordagens possíveis para reduzir a HP fixa em pacientes com ICFEr, os inibidores da fosfodiesterase-5, principalmente a sildenafila, são terapias bem estabelecidas e eficazes para pacientes com HP do grupo 1, isoladamente ou em combinação com outras terapias vasodilatadoras.^5^ Para a HP associada a ICFEr (grupo 2), estudos menores mostraram que a terapia com sildenafila melhorou a capacidade de exercício e a hemodinâmica pulmonar, mas estudos maiores sobre os resultados na ICFEr são escassos.^7,8^Um desses pequenos estudos randomizou 19 pacientes com ICFEr para uso de sildenafila 50 mg/3x/d versus placebo e também mostrou benefícios hemodinâmicos (diminuição dos níveis da pressão sistólica da artéria pulmonar em 4 semanas) no grupo tratado com sildenafila.^9^ A redução da RVP também foi demonstrada em um estudo unicêntrico de Pons et al.^10^ Quinze pacientes com uma RVP >2,5 unidades Wood foram tratados com sildenafila pré-TC com uma alta dose-alvo (109 ± 42 mg/dia) por 163 ± 116 dias. Benefícios importantes nas pressões pulmonares e na mortalidade pós-TC foram comparáveis entre o grupo com HP tratado e o grupo não grave, não tratado^10^ Em contraste, um ensaio multicêntrico de sildenafila em pacientes com ICFEp não conseguiu determinar os benefícios em relação ao estado clínico ou capacidade de exercício.^11^ Com base nesses achados preliminares positivos em pacientes com ICFEr, alguns centros de TC já adotaram o uso *off-label* de sildenafila como terapia de resgate em candidatos a transplante cardíaco selecionados, com HP fixa grave, com o objetivo de atingir um estado favorável ao transplante.^7^

O estudo^12^ agrega conhecimentos importantes a esse campo. Mendes et al.,^12^ compararam o efeito do tratamento com sildenafila pré-TC em 30 pacientes em relação a 205 pacientes com ICFEr não tratados com sildenafila. Esse foi um estudo observacional retrospectivo, unicêntrico, que comparou a hemodinâmica pulmonar e os desfechos clínicos em 1 semana e 1 ano após o TC em pacientes com HP fixa tratados por terapia com sildenafila 20 mg 3x/d a um grupo sem HP que não recebeu nenhum tratamento direcionado à HP. Apesar do desenho não randomizado, as características basais dos pacientes eram semelhantes, exceto pela gravidade da disfunção sistólica e a hemodinâmica da hipertensão pulmonar no grupo tratado com sildenafila. O estudo mostrou uma diminuição importante na pressão sistólica da artéria pulmonar e na resistência vascular pulmonar após uma prescrição de uso de sildenafila por 3 meses (58,9 a 52,8 mmHg e 5,4 a 3,3 unidades de Woods, respectivamente, com p <0,001 para ambas as análises). A melhora da hemodinâmica pulmonar levou a um benefício clínico significativo, o que possibilitou a elegibilidade da candidatura ao TC para aqueles 30 pacientes que foram, a princípio, considerados inelegíveis.

Apesar de haver pequenas diferenças na pressão sistólica da artéria pulmonar logo após o TC (7 dias), não houve diferenças significativas nas pressões pulmonares entre os grupos após 1 ano do transplante cardíaco, o que pode ter um impacto prático em relação a avaliações adicionais para outros pacientes com HP associada à IC na avaliação inicial para terapias avançadas. Resultados semelhantes foram demonstrados em uma pequena coorte de 18 pacientes que foram tratados com sildenafila para reduzir a HP associada à IC em uma tentativa de se tornarem elegíveis para o TC. Groote et al.^13^ relataram melhora significativa na classe funcional, fração de ejeção ventricular esquerda e parâmetros de teste de exercício cardiopulmonar, bem como uma redução considerável na resistência vascular pulmonar (de 5,3 ± 1,9 para 3,3 1,8 unidades Woods, p = 0,01).^13^ Outros estudos também demonstraram uma diminuição da pressão pulmonar em pacientes com HP persistente e dispositivos de assistência ventricular esquerda.^14^ Além disso, a sildenafila parece ser útil na insuficiência ventricular direita aguda e HP nas primeiras 72 horas após o TC em uma pequena série de casos de 13 pacientes.^15^

Embora tenha havido benefícios substanciais em relação aos parâmetros hemodinâmicos pulmonares, a mortalidade numericamente maior evidenciada no primeiro ano estava ligada à gravidade inicial da HP e deve ser levada em consideração no processo de tomada de decisão em relação à candidatura ao transplante cardíaco durante a avaliação inicial.

As limitações deste estudo estão relacionadas ao desenho metodológico observacional, retrospectivo, unicêntrico e ao número limitado de pacientes com HP incluídos. Além disso, a função ventricular direita não foi medida durante o estudo, o que pode ter prejudicado a interpretação hemodinâmica antes e após o tratamento com sildenafila. Embora este estudo provavelmente não tenha poder suficiente para tirar conclusões definitivas e validade externa, ele representa a maior coorte de pacientes com ICFEr grave tratados com sildenafila para iniciar um processo de TC.

Em conclusão, o tratamento com sildenafila em pacientes com ICFEr com HP grave possibilitou um período pós-operatório bem-sucedido em pacientes inicialmente considerados inelegíveis para TC, com melhora na resistência vascular pulmonar, mas sobrevida ligeiramente inferior 1 ano após o TC. Este é um achado importante para a prática clínica, pois pode indicar terapias avançadas inicialmente contraindicadas para candidatos a TC com HP grave associada à ICFEr, além de promover benefícios durante o período pré-TC.
