# Supplementary material for: Sildenafila como Terapia Adequada de Transplante Cardíaco para Insuficiência Cardíaca Avançada Associada à Hipertensão Pulmonar Fixa
Source: Arq Bras Cardiol. 2021 Feb 19;116(2):227–8. [Article in Portuguese] doi: 10.36660/abc.20200631 (PMC7909971; doi:10.36660/abc.20200631)
Supplement: Translation

# Translation: Sildenafil as an Eligible Heart Transplantation Therapy for Advanced Heart Failure Associated with Fixed Pulmonary Hypertension

**Authors:** Pedro Schwartzmann

**Affiliations:** 1 BrazilHospital Unimed Ribeirão Preto, Ribeirão Preto, SP - Brazil; 2 BrazilCentro de Pesquisa (CAPED) - Centro Médico RBS, Ribeirão Preto, SP - Brazil

**Keywords:** Heart Failure, Pulmonary Hypertension, Sildenafil Citrate/therapeutic use, Heart Transplantaion

Heart transplantation (HTx) has been associated to significant improvements in survival and quality of life in patients with advanced heart failure (HF). Despite a considerable increase in the indication of left ventricular assistant devices, HTx still remains the gold-standard approach in this clinical context.^1^ Even though it is considered the gold-standard treatment for advanced HF, several reasons have impaired the widespread utilization of the HTx. There is limited availability of donors compared with the growing potential recipients. Moreover, the longer life expectancy of the patients due to better HF pharmacotherapy has been challenging the age-related contraindication for the procedure, as well as the presence of more comorbidities linked to aging.^2^

Another point to be observed related to HTx candidacy is pulmonary hypertension (PH), which is present in more than 60% of HF patients with reduced ejection fraction (HFrEF) and over 54% of patients with HF and preserved ejection fraction (HFpEF) and also might account for up to 50% of post-HTx complications.^1^ An elevated pulmonary vascular resistance (PVR) > 2.5 Wood units is linked to a nearly 30% increase in mortality within the first-month post-transplant.^2^ This HF-associated PH is considered to be the result of the passive effect of increased left ventricular end-diastolic pressure along with vasoreactivity secondary to vasoconstriction and arterial pulmonary remodeling.^3,4^ When not reversible with a vasodilator challenge, it has been strongly associated with right ventricular dysfunction, HF hospitalizations, worsening in quality of life, and reduced survival.^5^ The International Society for Heart and Lung Transplantation guidelines consider the presence of severe pre-transplant PH as a relative contraindication to heart transplantation.^6^

Likewise, the disproportionality between the need for cardiac transplantation and the reduced availability of donors should lead to an even more judicious selection of those potential candidates for HTx and further attempts in reducing the pre-HTx pulmonary hypertension are necessary.

One of the possible approaches for lowering fixed PH in HFrEF patients, the phosphodiesterase-5 inhibitors, mainly sildenafil, are well established and effective therapies for patients with group 1 PH, either alone or in combination with other vasodilator therapies.^5^ For HFrEF-associated PH (group 2), smaller studies have shown that sildenafil therapy improved exercise capacity and hemodynamics, but larger trials on HFrEF outcomes are lacking.^7,8^ One of those small studies randomized 19 HFrEF patients to sildenafil 50 mg TID *versus* placebo and also showed hemodynamic benefits (decreased pulmonary artery systolic pressure levels at 4 weeks) in the sildenafil-treated group.^9^ The reduction in PVR has also been demonstrated in a single-center study by Pons et al.^10^ Fifteen patients with a PVR > 2.5 Wood units were treated with pre-HTx sildenafil with a high target dose (109 ± 42 mg/day) for 163 ± 116 days. Important benefits in pulmonary pressures and the post-HTx mortality were comparable between the PH-treated group and the non-severe, non-treated group.^10^ In contrast, a multicenter trial of sildenafil in HFpEF patients failed to determine benefits regarding clinical status or exercise capacity.^11^ Based on these positive preliminary findings in HFrEF patients, some HTx centers have already adopted the off-label use of sildenafil as a rescue therapy in selected heart transplantation candidates with fixed severe PH, aiming to achieve a transplant-favorable status.^7^

The study^12^ adds important knowledge to this field. Mendes et al.^12^ have compared the effect of pre-HTx sildenafil treatment in 30 patients to 205 non-sildenafil treated HFrEF patients. It was a retrospective, single-center, observational study that compared the pulmonary hemodynamics and clinical outcomes at 1-week and at 1-year after the HTx in patients with fixed PH treated with 20 mg TID of sildenafil therapy to a non-PH group that did not receive any PH-directed treatment. Despite the non-randomized design, the baseline patients’ characteristics were similar, but for the severity of the systolic dysfunction and the pulmonary hypertension hemodynamics in the sildenafil-treated group. The study showed an important decrease in the systolic pulmonary artery pressure and the pulmonary vascular resistance after a 3-month sildenafil prescription (58.9 to 52.8 mmHg and 5.4 to 3.3 Woods units, respectively, p<0.001 for both analyses). The pulmonary hemodynamics improvement led to a significant clinical benefit, which enabled the eligibility of HTx candidacy for those 30 who were, at first, considered to be ineligible patients.

In spite of having small differences in the systolic pulmonary artery pressure shortly after the HTx (7 days), there were no significant differences in the pulmonary pressures between the groups 1 year after the heart transplantation, which might have a practice impact regarding further assessments for other HF-associated PH patients on the initial evaluation for advanced therapies. Similar results were shown in a small cohort of 18 patients that were treated with sildenafil to reduce the HF-associated PH and try to become eligible for HTx. Groote et al.^13^ reported significant improvements in functional class, mean left ventricular ejection fraction and cardiopulmonary exercise testing parameters, as well as a considerable reduction in pulmonary vascular resistance (from 5.3 ± 1.9 to 3.3 ± 1.8 Woods units, p=0.01).^13^ Other studies have also shown a decrease in pulmonary pressures in patients with persistent PH and left ventricular assistant devices.^14^ Moreover, sildenafil seems to be useful for acute right ventricular failure and PH within the first 72 hours after HTx in a small case series of 13 patients.^15^

Although there were substantial benefits regarding the pulmonary hemodynamics parameters, the evidenced numerically higher mortality during the first year was linked to the initially PH severity and should be taken into consideration in the heart transplantation candidacy process decision-making during the initial evaluation.

Limitations of this study are linked to the retrospective, single-center, observational methodological design and the limited number of PH patients included. Also, right ventricular function was not measured during the study, which might have impaired the hemodynamics interpretation before and after sildenafil treatment. Although this study is unlikely powered for definitive conclusions and external validity, it might represent the largest cohort to date of severe HFrEF patients treated with sildenafil to enter a HTx process.

In conclusion, treatment with sildenafil in HFrEF patients with severe PH enabled a successful postoperative period for patients initially considered to be ineligible for HTx, with improvements in pulmonary vascular resistance but slightly lower survival 1 year after the HTx. This is an important finding for clinical practice as it might provide advanced therapies that are initially contraindicated for HTx candidates with severe HFrEF-associated PH, as well as promoting benefits on clinical status during the HTx waiting list period.
